# Hyalofast Cartilage Repair Surgery with a Full Load-Bearing Rehabilitation Program One Day after Operation Reduces the Time for Professional Athletes to Return to Play

**DOI:** 10.3390/medicina59040804

**Published:** 2023-04-20

**Authors:** Bartłomiej Kacprzak, Karolina Rosińska, Natalia Siuba-Jarosz

**Affiliations:** 1 Orto Med Sport Łódź, 28 Pułku Strzelców Kaniowskich 45, 90-640 Łódź, Poland; 2 Wolf Project Studio Krzysztof Król, ul. Gdańska 79/D01, 90-612 Łódź, Poland

**Keywords:** athletes, cartilage, Hyalofast, knee, membrane, microfracture, rehabilitation protocol, repair

## Abstract

*Background and Objectives*: This study evaluated the effectiveness of Hyalofast cartilage repair surgery with an early, full load-bearing rehabilitation program one day after the operation for reducing the time needed for professional athletes to return to play. *Materials and Methods*: This prospective study included 49 patients aged between 19 and 38 years who had undergone surgical reconstruction of cartilage using the microfracture technique combined with a Hyalofast scaffold. All patients were active professional athletes. Early rehabilitation was implemented from the first postoperative day, fully loading the operated limb. A clinical evaluation was based on the KOOS and SF-36 questionnaires used during subsequent follow-up visits. All patients underwent magnetic resonance imaging (MRI) to evaluate the effect of the surgery after one year. *Results*: The clinical results demonstrated a statistically significant improvement in the number of complaints about pain and in the quality of life of the patients, measured in all of the applied scales, with comparisons made between six months or one year post-surgery and pre-surgery. Importantly for athletes, the parameter related to sports and recreation improved from 14 ± 11.1 to 95 ± 7.7 6 months after surgery and to 99.8 ± 1.8 one year after surgery. The overall quality of life score improved from 30 ± 18 to 88 ± 8.8 one year after surgery. *Conclusions*: These results show that this approach significantly shortened the time needed for the athletes to return to sports at the same level as before the surgery (athletes returned to sports in approximately 2.5–3 months). The mean follow-up time was 19.75 months. This technique can be considered a viable option for the treatment of cartilage injuries in professional athletes, allowing them to return to play more quickly in a safe and healthy way.

## 1. Introduction

**Figure 1:**
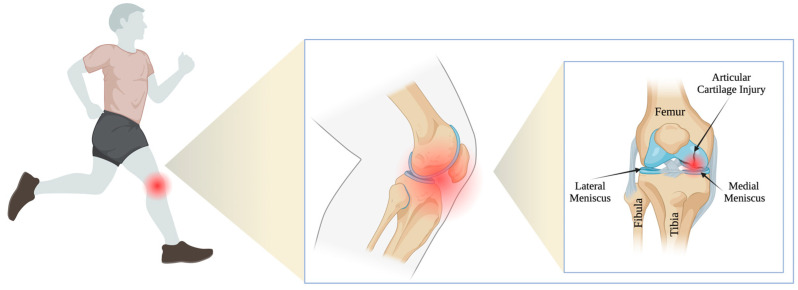
Articular cartilage injury in athletes.

The treatment of articular cartilage injuries has been a challenge for doctors around the world for decades. This is due to the fact that articular cartilage has limited healing potential due to a lack of blood vessels and nerve connections. Tissue nutrition depends mainly on the diffusion of nutrients from the joint cavity [1,2,3,4]. Articular cartilage damage is typically a long-term clinical orthopedic issue that has a huge impact on the functionality of people at any age [5,6]. The problem is so widespread that it currently affects more than 50 million American adults, and it is estimated that this number is expected to rise to approximately 67 million by 2030 [5,7]. Intra-articular injuries, potentially leading to degenerative changes, are much more common in the case of professional athletes [8]. Epidemiological data suggest that athletes suffering from acute injuries and experiencing long-term stress on their joints related to rapid turns and changes of direction reveal symptoms of cartilage damage and early degenerative changes more frequently [9,10] (Figure 1). Physical activity through participation in organized team sports plays an important role in maintaining joint cartilage health. At the same time, it is also beneficial in limiting the progression of osteoarthritis (OA) [11,12,13]. However, participation in some sports may increase the risk of knee OA, which has been reported in contact and collision sports (e.g., soccer [14,15,16] and rugby [17]).

Current therapeutic solutions are aimed at reducing cartilage load and promoting the cartilage regeneration process [18,19,20,21,22]. Conservative treatments often include load correction with orthoses, weight loss, the use of special orthotic insoles and shoes, and even walking with orthopedic crutches [23]. The ineffectiveness of conservative treatments in high-grade cartilage injuries, the increasing number of such injuries, and athletes’ need to return to sports quickly all drive physicians and researchers to look for alternative surgical methods of treatment. Some of the surgical treatment methods used in clinical practice are, autologous chondrocyte transplants [24], microfractures [25,26,27], osteochondral transplants [28], and scaffolds [29,30,31,32]. Scaffolds in tissue engineering are functional substitutes for bone defects. In orthopedic applications, placing such scaffolds directly at the site of injury promotes the process of bone healing [33]. Scaffolds used in bone tissue engineering can be constructed from various materials, such as metals (e.g., biodegradable magnesium [34]), synthetic polymers (e.g., PCL [35,36]), or natural materials (e.g., collagen, hyaluronic acid [29,30,31,32,37,38]). For cartilage tissue regeneration, scaffolds made of natural materials are preferred as high mechanical strength is not required, unlike in bone reconstruction. In this research, a systematic review showed that early microfracture treatment of cartilage defects is associated with positive clinical and histological outcomes [39,40].

Bone marrow stimulation techniques (such as microfracturing) have been used since the 1950s, and were originally developed by Pridie and Steadman when they tried to induce subchondral bone bleeding to release multipotent mesenchymal stem cells and growth factors into the defect site of cartilage, leading to cartilage repair [41,42]. The main advantage of this treatment technique over grafts is that it avoids infection and health problems at the donor site, and it reduces the risk of the need for further surgery. However, the resulting clot has an inferior ability to withstand repeated load-bearing. For this reason, biological scaffolds consisting of a matrix of porcine collagen, polyglycolic acid, or hyaluronic acid are additionally placed over the cartilage defect after microfracture during surgery, creating a mechanically stable structure into which the subchondral blood soaks. The inserted matrix thus acts as a biocompatible and temporary scaffold that concentrates mesenchymal stem cells and growth factors only at the defect site, rather than throughout the joint [42,43,44].

The repair of cartilage defects with a Hyalofast^TM^ membrane—a fibrin substrate that stimulates the cartilage to grow—has proven to be an interesting alternative for patients with intensive joint pain [45,46,47,48,49]. Hyalofast is a white fibrin substrate, composed entirely of a semi-synthetic derivative of hyaluronic acid which is a natural component of the extracellular matrix and the main component of human cartilage. It allows for the differentiation of mesenchymal stem cells into chondrocytes, which are able to produce physiological cartilage. It forms a chondroprotective layer that extends the survival of mesenchymal stem cells in situ after mobilization by the microfracture technique [47,48,49,50,51].

However, it should be noted that despite significant advances in the treatment of cartilage damage, recovery can be prolonged, significantly delaying the return to sports for athletes. Therefore, the preparation of an appropriate rehabilitation program is essential to optimize the results of the surgical treatment of cartilage injuries. Rehabilitation protocols can significantly improve the cartilage repair process, thereby increasing athletes’ physical activity and reducing the risk of re-injury [4]. This is because such surgeries often require a significant amount of time for the knee to fully heal and regain its strength and stability. A well-designed rehabilitation program can not only facilitate the healing process, but can also ensure that the athlete is able to return to their sport at full capacity and with reduced risk of re-injury. It is known that physical exercise increases bone and muscle mass, while periods of inactivity are associated with tissue atrophy. There is evidence that cartilage atrophy occurs under conditions of reduced loading, which may occur in cases of prolonged postoperative immobilization [52]. Previous studies support the early resumption of a full range of motion to improve cartilage healing [53,54,55,56]. Animal studies using continuous passive motion have shown that earlier resumption of a full range of motion results in improved chondrogenesis, proteoglycan, and glycosaminoglycan synthesis in cartilage, as well as reduced collagen breakdown [54,56,57]. In a review conducted by Hurley, it was shown that rehabilitation protocols associated with full weight bearing on the treated joint in individual studies are highly variable, with most protocols allowing partial weight bearing within the first month [56]. Early rehabilitation may be beneficial from a psychological standpoint post-surgery, as it allows for earlier return to normal daily activities and encourages a gradual increase in activity [58]. In all procedures presented in Hurley’s work, the most common time point for allowing full weight bearing was six weeks [56]. However, this potential benefit must be balanced with protection of the site of cartilage regeneration, as earlier rehabilitation may overly stress this area and cause early failure. It is still unclear from the literature which time point is safest to allow full weight bearing on the treated joint with a full range of motion. Protocols and return-to-play criteria after knee joint cartilage reconstruction surgeries are highly variable.

The objective of this study was to investigate the effectiveness of arthroscopic surgery using the microfracture technique combined with a Hyalofast scaffold to treat knee cartilage injuries followed by an intensive rehabilitation program with full load bearing just after surgery among professional athletes. This article highlights the novelty of a full weight-bearing approach for the treated joint very shortly after cartilage reconstruction surgery, along with an intensive rehabilitation program. This approach is supported by MRI evidence, and the KOOS and SF-36 questionnaire results suggesting that early resumption of a full range of motion can improve cartilage healing. It also emphasizes the importance of individualized rehabilitation protocols that consider the patient’s specific condition and progress, as well as close monitoring by a healthcare professional. Compared to the literature data on rehabilitation programs, the proposed treatment approach allows patients to have full range of motion with full weight bearing without the use of orthoses as early as one day after surgery (under continuous monitoring by attending physicians). The combination of full weight bearing and intensive rehabilitation may offer a promising approach to enhance postoperative recovery and optimize the required recovery time for athletes undergoing cartilage reconstruction surgery (reducing the required time needed to return to sports at pre-injury level to 2.5–3 months).

## 2. Materials and Methods

### 2.1. Patient Selection

**Figure 2:**
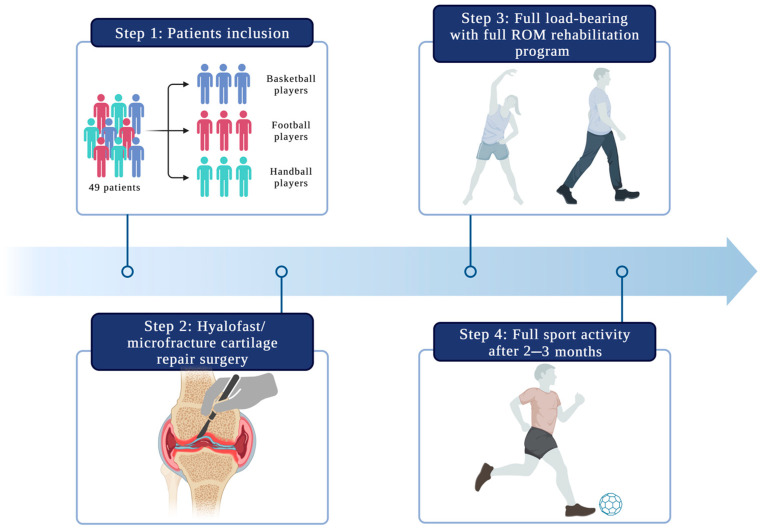
A proposed therapeutic approach for articular cartilage injuries in high-performance athletes.

The patients were classified for the procedure according to the ICRS classification. In total, 49 patients, 34 men and 15 women aged 19–38 years (mean = 30 years), underwent cartilage reconstruction surgery. All of these patients were active professional athletes (36 football players, eight basketball players, and five handball players—3 sports disciplines that expose athletes to pivoting/rotational injuries) (Figure 2). Twenty-nine patients presented lesions on the lateral femoral condyle (LFC; 59.2%). The study included patients with a height of 170–185 cm and a weight of 60-70 kg. Twenty patients had lesions on the medial femoral condyle (MFC; 40.8%). Nineteen patients underwent an additional partial or total meniscectomy during surgery. The most common causes of cartilage damage in patients included trauma (65%), osteochondritis dissecans (OCD; 22%), and non-traumatic causes (13%). The mean follow-up time was 19.75 months (SD = 6.79, range = 14–24 months). Table 1 presents information on the patients included in this study.

The inclusion criteria of the study included active athletes with grade IV articular cartilage focal damage who were indicated for surgery and who did not have a history of cartilage surgery on the affected knee joint. The exclusion criteria included patients under 18 years old or above 50 years old, patients with muscle disorders such as myasthenia gravis, progressive malnutrition, or periodic paralysis. Patients who were not willing to cooperate with the treatment, as well as those who refused to rehabilitate under supervision at our clinic, were also excluded from this study. It was also impossible to include patients who had abnormal bleeding or an abnormal coagulation function and patients with incomplete follow-up and imaging data or a follow-up time of less than six months.

The patients underwent cartilage regeneration surgery with the use of a Hyalofast membrane (Anika Therapeutics Inc., Bedford, MA, USA), microfractures, and tissue glue stabilization. After an MRI evaluation of the lesion size and location, a physical examination, an assessment of pain intensity, and verification of the failure of conservative treatment, the patients were referred for surgery by their attending physician. The study protocol was approved by the local ethics committee of the authors’ affiliated institution. All patients gave their written informed consent to participate in the study.

### 2.2. Operation Procedure

**Figure 3:**
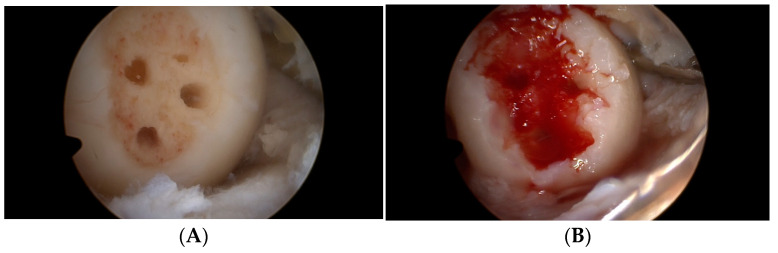

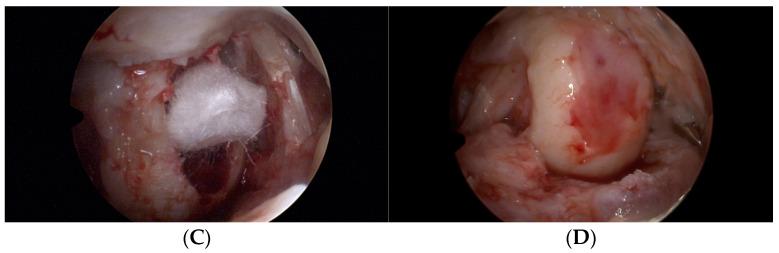
The site of the cartilage defect. Microfractures were made in the bottom (**A**), filled with the patient’s blood (**B**), covered with a Hyalofast membrane (**C**), and then stabilized with a tissue adhesive (**D**).

The same team and surgeon performed all activities under general anesthesia. All procedures involving the knee joint were performed arthroscopically. The first step of surgery involved the insertion of the two classic anteromedial and anterolateral portals and an accurate assessment of the joint structures—ligaments, menisci, and type and depth of cartilage damage. In those patients with meniscal tears requiring removal, a partial or total meniscectomy was performed. After a careful cleaning of the lesion with the complete removal of damaged tissue, the size of the cartilage defect was assessed. Then, microfracturing was performed at the base of the defect using a dedicated set of tools (Figure 3A). Following this, the base of the defect was filled with the patient’s blood (Figure 3B). The site was then cleaned to ensure good visibility for implantation of the Hyalofast scaffold (Figure 3C). A membrane sized to match the defect area was cut from a 2 × 2 cm or 5 × 5 cm Hyalofast scaffold, and was then inserted by a cannula into the joint and placed in the defect with the aid of a needle and a probe. After stabilization of the scaffold at the defect, it was re-filled with the patient’s blood. TISSEEL Lyo tissue glue (Baxter International Inc., Deerfield, IL, USA) was then used for the final fixation of the Hyalofast membrane (Figure 3D). The patients received anticoagulants (Neoparin 0.4/0.6 for 20 days once a day; SCIENCEPHARMA SP. Z O.O. SP.K., Warsaw, Poland) and antibiotic therapy for two weeks after surgery (Clindamycin 600, every 8 h; MIP PHARMA POLSKA SP. Z O.O., Gdańsk, Poland). No drains or joint punctures were used at any phase of the therapy.

### 2.3. Follow-Up Evaluation

We evaluated the treatment results comparing the patients’ preoperative situation with a functional assessment performed during the follow-up visits. The patients completed the SF-36 and KOOS questionnaires before surgery and at 2, 6, 12, and 24 weeks and 1 year after surgery. The Knee Osteoarthritis Outcome Score (KOOS) is composed of five subscales that assess pain severity (PAIN), symptoms (SYMPTOMS), function in daily living (ADL), function in sports and recreational activities (SPORT/REC), and quality of life (QOL) [59]. The final tool used for the subjective assessment of the patients’ general health and well-being was the SF-36. This contains eight domains used to assess both physical and mental functioning [60]. Furthermore, we assessed the cartilage regeneration process by 1.5 T magnetic resonance imaging (SIGNA 1.5T HDx; GE HealthCare Technologies Inc., Chicago, IL, USA) scans (on average, after six to eight weeks, six months, and one year). MRI allows multidimensional evaluation of the joint; it is highly sensitive to soft tissue, enabling an accurate evaluation of the cartilage and its remodeling process [49,61].

### 2.4. Rehabilitation Protocol

No brace was used, nor was any stabilization. After the operation, oedema and pain were controlled by a Game Ready cooling system and compression stockings (GAME READY knee wrap (CoolSystems, Inc., Alpharetta, GA, USA) and a regeneration and massage system (Normatec (Hyper Ice, Inc., Irvine, CA, USA)). A physiotherapy program was started on the first day after the operation, including walking on the operated limb with a full load, supported by elbow crutches. The crutches were put away seven days after the operation. The patients were not limited in their range of movement after surgery. The physiotherapy protocol included resistance training, eccentric–concentric exercises, and exercises with full body weight, with a focus on force and increasing the movement range of the erector and flexor muscles of the knee (AlterG antigravity treadmills (AlterG, Inc., Fremont, CA, USA) and HydroWorx underwater treadmills (HydroWorx, Middletown, PA, USA). The patients learned to carry out normal movement patterns under supervision. A strong emphasis was constantly placed on proprioception exercises under unstable ground conditions. The exercise package was completed by the manual activities of a physiotherapist. By three weeks after the surgery, the athletes had already started preliminary training sessions under the supervision of a physiotherapist and a physician. Additionally, 02 Prime Hyperbaric Chambers (HearMEC Co., Ltd., Tokyo, Japan) and athletic compression sportswear (CEP, Bayreuth, Germany) were also used during rehabilitation program. The rehabilitation room undergoes regular sanitary-epidemiological inspections. Due to its nature as a medical centre, each piece of equipment has a current mandatory technical passport that is renewed once a year. Each patient is guided by the same therapist throughout the entire treatment process, and the same person operates the rehabilitation equipment.

### 2.5. Statistics

The obtained results are presented as the mean value ±standard deviation (SD). All statistical data were analyzed using STATA software (Stata, version. 12.0; StataCorp, College Station, TX, USA). Differences were compared using an unpaired *t*-test or a Mann–Whitney *U*-test for continuous data, while for categorical data, a Pearson’s chi-square test was used. A *p*-value of less than 0.05 was considered significant.

## 3. Results

### 3.1. KOOS and SF-36 Scales

The patient evaluation results from two questionnaires (KOOS and SF-36) are presented below. Table 2 and Table 3 show the overall results for all patients, while Figure 2 presents graphs with data for each group of athletes (basketball, football, and handball).

The obtained results from the KOOS and SF-36 questionnaires show a significant increase in the patients’ health status and quality of life as soon as six months after surgery. On the KOOS scale, pain improved from 55 ± 18.2 to 96 ± 4.2 by the sixth month and to 99.5 ± 2.3 by the 12th month. The symptoms improved from 46 ± 16.6 to 93 ± 6.5 by the sixth month and to 99.5 ± 2.3 by the 12th month. The parameter for activities of daily living increased from 57 ± 25.5 to 100 ± 0.0 one year after surgery. Importantly for athletes, the parameter related to sports and recreation improved from 14 ± 11.1 to 95 ± 7.7 by six months after surgery and to 99.8 ± 1.8 one year after surgery. The overall quality of life score improved from 30 ± 18 to 88 ± 8.8 one year after surgery. There was a statistically significant difference in the KOOS scores evaluated before and at 6 and 12 months after surgery. A statistically significant difference was noted in the following SF-36 subscale scores at six months postoperatively: bodily pain improved from 36 ± 18.7 to 78 ± 15.6 and physical function from 34 ± 18.5 to 99 ± 2.0. One year after surgery, a statistically significant difference was noted for all SF-36 parameters.

**Figure 4:**
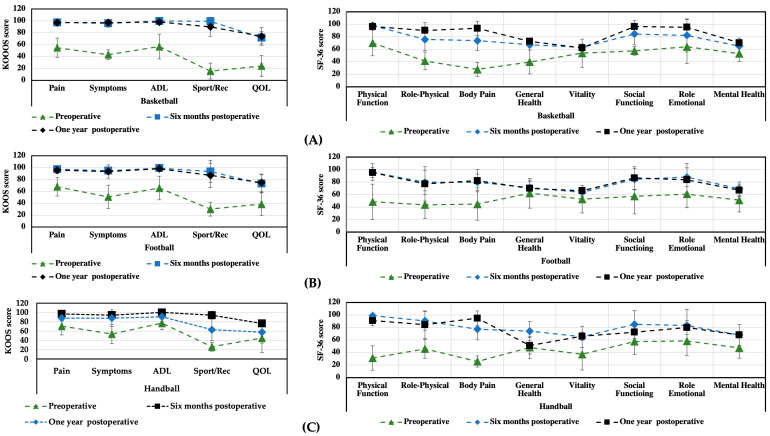
Comparison of the preoperative and postoperative results from the KOOS and SF-36 questionnaires for different groups of athletes: basketball players (**A**), football players (**B**), and handball players (**C**).

The results from a comparison of the KOOS and SF-36 scores for different sports indicate the best progress for the basketball and football players (Figure 4). For most parameters, the scores remained similar for 6 and 12 months after surgery (or an increase was observed with increasing recuperation time). For the handball players, the postoperative scores from both scales indicated progress compared to the preoperative scores; however, in the case of the SF-36 scale, some parameters (e.g., physical function and general function) were observed to get worse.

**Figure 5:**
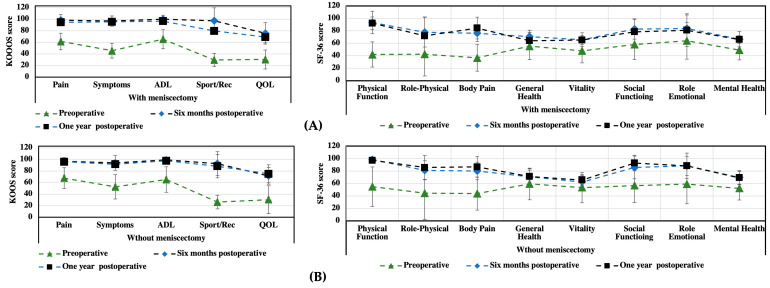
Comparison of the preoperative and postoperative results from the KOOS and SF-36 questionnaires for patients undergoing concurrent treatment of meniscal lesions (**A**) and patients treated only for chondral lesions (**B**).

An additional analysis was performed to determine the difference between those patients who underwent additional treatment of their meniscal lesions during the treatment of their cartilage injuries and those patients treated solely for chondral changes (Figure 5). The SF-36 results did not show any significant differences between these two groups; however, the KOOS results indicated better outcomes for the Sport/Rec and QOL parameters for those patients without additional treatment for meniscal lesions (treated solely for chondral changes). It should be emphasized, however, that in both analyzed groups, the treatment method used resulted in a significant improvement in the patients’ health condition.

### 3.2. Functional Assessment

Overall, three to four weeks after the surgery, all of the patients returned to activities of daily living without pain symptoms. The time of return to sports varied and ranged from two to four months. All professional athletes (100%) returned to practicing their sporting discipline at their pre-injury level (mean time = 2.5–3 months). At the 6- and 12-month follow-up visits, we did not observe any abnormalities in terms of range of motion, oedema, or pain in the treated joint.

### 3.3. Radiological Evaluation

**Figure 6:**
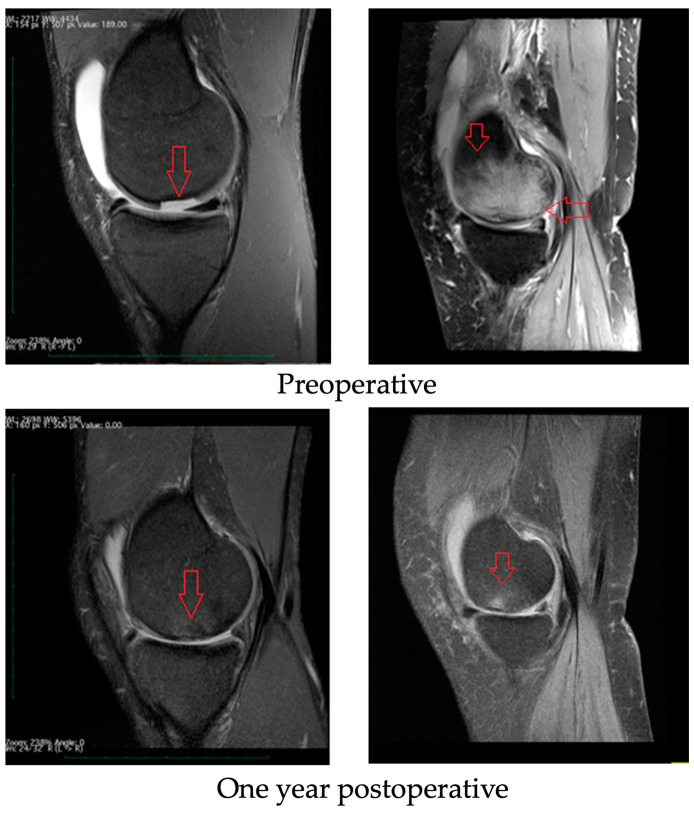
MRI findings: preoperative and one year postoperative. Red arrows for preoperative images show cartilage damage with ICRS grade IV loss (image on the left) and marrow oedema as a result of cartilage damage and lack of treatment (image on the right) and for one year postoperative images red arrows show evolution of cartilage healing (image on the left) and withdrawal of oedema from bone (image on the right).

All patients underwent a postoperative SIGNA 1.5T HDx MRI scan. Similar results were obtained for all groups of athletes included in this study. An example of the results is shown in Figure 6. Comparing the pre- and postoperative MRI scans, it is noted that the periosteal inflammatory reaction and bone marrow oedema resolved, and the cartilage defects were filled with new tissue. Before the operation, fluid could be seen in the supraspinatus lobe (which is the result of damage and overloading of the knee). After surgery, the fluid began to be absorbed and did not recur after exercise. The oedema of the subchondral layer (visible in the form of light-colored bone) retreated (dark-colored bone one year after surgery), indicating that the wound was healing.

### 3.4. Complications

No complications were found after surgery, such as infection, bleeding, liver/kidney dysfunction, or other knee injury.

## 4. Discussion

Several techniques for the surgical treatment of cartilage defects have been developed in recent years. The choice of the most appropriate method is still controversial. Arthroplasty is one of the most common solutions described in the literature and used in clinical practice for the treatment of high-grade chondral defects [50]. It is a highly invasive and expensive procedure. Since the lifespan of an implant is approximately 15–20 years, it represents a solution for people over 60 years of age. Our study showed that there is a valid alternative treatment. As is evident from MRI scans (Figure 6), even large lesions involving practically the entire medial femoral condyle presenting grade IV cartilage damage are reversible with the use of appropriate therapeutic management. In our work, we showed that Hyalofast scaffold implantation combined with the microfracture technique, followed by a proper, supervised rehabilitation protocol, is an effective method for treating chondral tissue defects, as evidenced through pre- and postoperative MRI scans. Based on the subjective SF-36 and KOOS scores, we observed a marked improvement in the patients’ comfort and quality of life, as well as an improvement in physical function. Moreover, this method is a good option for professional athletes who wish to return to sports as soon as possible and to maintain the highest possible articular function for many years to come. The proposed treatment, combined with the author’s rehabilitation program, can be successfully applied to athletes from various sports (basketball, football, and handball). From the perspective of athletes, continuous rehabilitation and training are very important. Otherwise, by not following the physician’s recommendations, their well-being can decline, as noted in the case of handball players.

The use of the Hyalofast scaffold has showed positive results in other research. Quiceno conducted a study to evaluate the short- and medium-term outcomes of patients treated with autologous matrix-induced chondrogenesis (AMIC) using a hyaluronic acid-based scaffold with grade IV cartilage changes in the knee. The results of this study indicated that patients who underwent this procedure demonstrated satisfactory results throughout the observation period [62]. In Gobbi’s work, the effectiveness and regenerative capacity of autologous adult mesenchymal stem cells with a hyaluronan-based scaffold (Hyalofast) in treating grade IV cartilage changes in the knee joint in patients over 45 years of age were subjected to a medium-term evaluation. Based on the results obtained, it was demonstrated that the proposed treatment method is an effective option regardless of age [63]. In another study, Gobbi also showed that repairing knee joint cartilage damage using a hyaluronic acid-based scaffold with bone marrow aspirate concentrate (HA-BMAC) provides good or excellent clinical outcomes in long-term observation for treating small and large lesions. This method of treatment provides comparably good long-term results for small or large, single or multiple lesions [64]. A paper published by Tan et al. also supports our observations [65]. The authors showed, in a group of 46 patients with grade IV knee cartilage damage, that the use of Hyalofast combined with the microfracture technique is a feasible treatment alternative. In their study, all KOOS subcategory scores assessed one, two, and three years after surgery improved significantly in comparison to the preoperative scores. The authors also presented postoperative MRI scans, showing cartilage defects filled with chondral tissue, and with almost complete repair of the cartilage surface.

The data available in the literature demonstrate that use of the microfracture technique to improve the function of affected joints [66,67] is associated with a good short-term effect. Microfractures stimulate the bone marrow to produce collagen fibers, but not the proper type of collagen for articular cartilage (collagen type II) that is used to construct the articular surface. Microfracturing brings about the formation of fibrocartilage, a sort of scar tissue of the cartilage that does not last in the joint, since it does not have the mechanical properties of the original hyaline cartilage [68]. Yen et al. also pointed out the vital role of the rehabilitation process in the final treatment outcome. However, their rehabilitation protocol was less aggressive than the one adopted in our study [69]. Mithoefer et al., in their study, presented a group of 48 patients with full-thickness knee cartilage defects that were treated with microfracturing alone [70]. All parameters covered by the SF-36 quality of life score improved; 67%, 25%, and 8% of patients achieved very good, good, or poor knee function scores, respectively. On the postoperative MRI scans, 13 (54%), 7 (29%), and 4 (17%) patients obtained good, moderate, and poor defect filling, respectively. Such results (almost complete defect filling) depend not only on the use of the microfracture technique itself, but also on its combination with Hyalofast.

As the problem of cartilage damage affects many professional athletes, physicians are faced with the duration of the recovery period and with the possibility of regaining the pre-injury performance level. Della Villa et al., in their study, suggested the need to delay the moment when the treated joint can be fully loaded [3]. They justified their opinion by the fact that autologous chondrocyte implantations (ACIs; cell grafts that require time to mature and create new cartilage) were used. Therefore, the time at which the treated limb is allowed to be fully weight bearing is postponed until three to four weeks after the procedure. Meanwhile, work prepared by Hambly’s team suggests that the time taken for graft remodeling is at least 18 months, when the cartilage becomes more hyaline. In this regard, the authors suggested prolonging the return-to-pitch time from 12 to 18 months [71]. In modern professional sports, such an extended time period is considered too long and may result in athletes missing one or even two seasons of play. Thus, our treatment approach, which eliminates this long recovery time before the treated joint is allowed to be fully weight bearing, seems to be much more attractive. Nevertheless, Della Villa confirmed that an intensive rehabilitation program enables a better therapeutic effect to be obtained with a faster and safer return to sports [4].

In a systematic review published in 2009, Mithoefer et al. assessed the recovery time and the percentage of professional athletes returning to active sports after articular cartilage damage [72]. A return to sports was possible in 73% of cases, and autologous osteochondral implantations provided the best outcomes. The time needed to return to sports ranged from 7 to 18 months, depending on the technique used to repair the cartilage damage. It was shown that returning to sporting activity depended on the following factors: the athlete’s age, duration of the symptoms before surgery, level of sports performance, size of the defect, and the surgical technique used. Another important factor affecting the efficiency of reconstructed intra-articular injuries is the patient’s body mass (body mass index), as demonstrated, for example, in the case of the hip joint [73]. However, in this study, patients with high levels of physical activity, in whom body mass index did not have a significant impact on the rate of cartilage regeneration, were treated (average mass of 60–70 kg and average height 170–185 cm). In this case, an appropriate rehabilitation program proved to be crucial. Our study showed that, regardless of the above factors, it is possible to obtain a positive therapeutic effect when all of the following elements of the procedure are used: Hyalofast scaffold, microfracture technique, tissue glue, and full cooperation between physiotherapists, patients, and physicians during the rehabilitation process.

Our treatment option is unique in comparison to the published literature. Some other authors have suggested that weight bearing on the limb should be restricted for a period of four, six, and even eight weeks and indicate the need for a range of motion (ROM)-limiting orthosis. In comparison to this approach, early mobilization and early full weight bearing of the treated joint (the first day after surgery), with the use of crutches being stopped two weeks later, seems to create quite a radical approach. Such a procedure, as evidenced by our paper, not only does not impact the cartilage regeneration process adversely, but it enables the patient to rapidly regain sporting activity and independence. In general, one to two weeks after surgery the patient can function in their daily life without pain. In all patients presented, elimination of pain was sustained with no recurrence. Three weeks after surgery, the patients started a pitch training program adapted to the sporting discipline practiced. This is a crucial element in preparing the patient to return to sports and to team training, which we achieved by preventing patients from developing a fear of re-injury and by increasing their confidence in the use of the treated limb. The longest follow-up period was 24 months. During that period, we did not observe any treatment failure or recurrence of symptoms within the treated knee joint. Moreover, MRI scans showed, in all cases, the healing of the defects with regenerated cartilage and elimination of the periosteal reaction.

The limitations of our study include not having a control sample treated with a less-intensive rehabilitation program. However, in reality, patients who are not active athletes usually do not show such motivation during the rehabilitation period. Additionally, conducting a randomized trial with a longer follow-up period would provide more observations, but such a study may be difficult to conduct.

## 5. Conclusions

In this work, the effectiveness of knee cartilage defect treatment was analyzed. Hyalofast membranes, combined with microfracture surgery and a tissue adhesive, supported by an intensive postoperative physiotherapy protocol, significantly improved the quality and comfort of life of the operated patients. Compared to the preoperative and follow-up period, the proposed treatment procedure allows a significant reduction in the time needed for the patient to return to normal and sporting activities, showing excellent results for athletes. The implementation of an intensive postoperative rehabilitation program is key to optimizing the results of cartilage defect repairs and influences the time needed to return to full athletic performance. Based on the conclusions of the present study, there are several areas for future research on knee cartilage defect treatment. Firstly, additional randomized trials could be conducted to confirm the findings of the current study and to assess the long-term outcomes of the proposed treatment procedure. Further investigation could also examine the effectiveness of the proposed treatment procedure in different patient populations, such as older individuals or those with more severe cartilage damage. Secondly, future research could focus on identifying the optimal international postoperative rehabilitation protocol for patients undergoing knee cartilage defect treatment and also other injuries (e.g., ACL injuries). This could involve exploring different exercise programs or evaluating the use of other therapeutic modalities, such as those proposed in this article.

## Figures and Tables

**Table 1:** Characteristics of the athletes included in this study.

	Knee Joint (*n* = 49)
Age, mean ± SD	30 ± 6.7
Sporting activity	
Professional	49
Gender, F/M	15/34
Cause of damage	
Traumatic	32
Non-traumatic	6
OCD	11
Location of the lesion	
LFC	29
MFC	20
Size of lesion (cm^2^), mean ± SD	2.96 ± 1.05
Previous surgeries	4 ACL reconstructions
Accompanying procedures	
Meniscectomy	19

**Table 2:** Comparison of the preoperative and postoperative results from the KOOS questionnaire for all patients.

	Preoperative	Six Months Postoperative	One Year Postoperative	(before/after Six Months)
KOOS	Mean ± SD	Mean ± SD	Mean ± SD	*p*
Patients (*n*)	49	49	43	
Pain intensity	55 ± 18.2	96 ± 4.2	99.5 ± 2.3	<0.05
Symptoms	46 ± 16.6	93 ± 6.5	98.2 ± 1.8	<0.05
Activities of daily living	57 ± 25.5	99 ± 2.6	100 ± 0.0	<0.05
Sport/Rec	14 ± 11.1	95 ± 7.7	99.8 ± 1.8	<0.05
Quality of life	30 ± 18	76 ± 4.7	88 ± 8.8	<0.05

Abbreviations: SD, standard deviation; *p* = statistical significance (*p* < 0.05).

**Table 3:** Comparison of preoperative and postoperative results from SF-36 questionnaires for all patients.

	Preoperative	Six Months Postoperative	One Year Postoperative	
SF-36	Mean ± SD	Mean ± SD	Mean ± SD	(before/after Six Months)
Patients (*n*)	49	49	43	*p*
Physical function	34 ± 18.5	95.6 ± 2.0	97 ± 2.5	<0.05
Role physical	63 ± 46.5	76 ± 15.0	96 ± 3.7	0.345
Bodily pain	36 ± 18.7	78 ± 15,6	98 ± 2.3	<0.05
General health	68 ± 17.1	70 ± 16.1	83 ± 15.8	0.401
Vitality	53 ± 20.6	64 ± 8.0	87 ± 7.1	0.067
Social function	64 ± 29.6	87 ± 13.6	90 ± 9.5	0.068
Role emotional	73 ± 42.0	83 ± 22.9	90 ± 13.3	0.285
Mental health	52 ± 9.8	68 ± 11	70 ± 10.8	0.464

Abbreviations: SD, standard deviation; *p*, statistical significance (*p* < 0.05).

## Data Availability

The data presented in this study are available on request from the corresponding author. The data are not publicly available due to patient data protection.
